# Classification of esophageal cancer by using hyperspectral data

**DOI:** 10.1007/s11548-025-03514-x

**Published:** 2025-09-23

**Authors:** Marianne Maktabi, Claudia Hain, Hannes Köhler, Benjamin Huber, René Thieme, Katrin Schierle, Boris Jansen-Winkeln, Ines Gockel

**Affiliations:** 1 Department of Electrical, Mechanical and Industrial Engineering, Anhalt University of Applied Science, Köthen (Anhalt), Germany; 2 Innovation Center Computer-Assisted Surgery (ICCAS), University of Leipzig, 04103 Leipzig, Germany; 3 Department of General and Visceral Surgery, SRH Klinikum Burgenlandkreis GmbH, 06712 Zeitz, Germany; 4 Institute of Pathology, University Hospital Leipzig, 04103 Leipzig, Germany; 5 Department of Visceral, Transplant, Thoracic and Vascular Surgery, Faculty of Medicine, University of Leipzig, Leipzig, Germany; 6 Department of General, Visceral, Thoracic and Vascular Surgery, Clinic St. Georg Leipzig, Leipzig, Germany; 7 Department of Visceral, Vascular and Emergency Surgery, Klinikum Magdeburg gGmbH, 39130 Magdeburg, Germany

**Keywords:** Hyperspectral imaging (HSI), Hyperspectral data, Artificial intelligence (AI), Esophageal cancer, Gastric cancer, Near-infrared perfusion index (NIR-PI), Esophagogastric surgery

## Abstract

**Purpose:**

Esophageal cancer is widespread worldwide, with the highest rate in Asia. Early diagnosis plays a key role in increasing the survival rate. Early cancer detection as well as fast evaluation of tumor extent before and resection margins during/after surgery are important to improve patients’ outcomes. Hyperspectral imaging (HSI), as a noninvasive and contactless novel intraoperative technique, has shown promising results in cancer detecting in combination with artificial intelligence.

**Methods:**

In this clinical study, the extent to which physiological parameters, such as water or hemoglobin content, differ in the esophagus, stomach, and cancer tissue, was examined. For this purpose, hyperspectral intraluminal recordings of affected tissue specimen were carried out. In addition, a classification of the three intraluminal tissue types (esophageal, stomach mucosa, and cancerous tissue) was performed by using two different convolutional neural networks.

**Results:**

Our analysis clearly demonstrated differences in hemoglobin concentration and water content between healthy and cancerous tissues, as well as among different tumor stages. As classification results, an averaged area under the curve score of 81 ± 3%, a sensitivity of 74 ± 8%, and a specificity of 89 ± 2% could be achieved across all tissue types using a hybrid convolutional neural network.

**Conclusion:**

HSI has relevant potential for supporting the detection of tumorous tissue in esophageal cancer. However, further analyses including more detailed histopathologic correlation as “gold standard” are needed. Data augmentation and future multicenter studies have to be carried out. These steps may help to improve and sharpen our current findings, especially for esophageal cancerous tissue.

## Introduction

Esophageal cancer ranks seventh among the most frequently diagnosed cancers and holds the sixth position among the leading causes of cancer-related deaths worldwide [1]. Diagnosis, treatment, and severity of esophageal cancer largely depend on its type and tumor stage at first detection. Two distinct histopathologic entities of esophageal tumor types are the most common: Esophageal Squamous Cell Cancer (ESCC) and Esophageal Adenocarcinoma (EAC). In the upper and middle third of the esophagus, the carcinomas are predominantly ESCC. In the lower third of the esophagus and at the esophagogastric junction (EGJ), EAC occurs almost exclusively [2, 3].

Adenocarcinomas with the predominating tumor mass at the area of the Z-line are independently classified as AEG (adenocarcinomas of the esophagogastric junction) [4], and are divided into three subtypes according to Siewert [2]. Siewert type I is formally considered as distal EAC. AEG type III by biologic tumor behavior acts as a “classical” gastric cancer [5]. Siewert type II is the actual cardia carcinoma and could be either treated surgically as AEG type I or as type III [6]. Increase in global obesity and gastroesophageal reflux disease (GERD) is associated with a rising number of EAC cases, especially with AEG as compared to ESCC [7–9]. Due to the late onset of symptoms and a high risk of early lymphatic spread, even at a low tumor infiltration depth [10], first diagnosis is often performed at an advanced tumor stage only, with lymphatic and vascular tumor infiltration already present [1]. Despite advances in diagnostics and therapy, the prognosis of esophageal cancer still remains poor [11].

Therefore, particular attention has to be paid to the early detection of these tumors. Currently, the “gold standard” for diagnosis is esophagogastroscopy with biopsy, followed by histopathological analysis [12]. However, it is invasive, carries reasonable risks and potential complications, and small lesions as precursors can be overlooked easily. Thus, there is a large interest in making early lesions optically better visible [13]. In addition, a reliable procedure is required to assess the extent of the resection before and the resection margins during/after endoscopic or surgical removal of the lesion. Hyperspectral imaging (HSI) offers a contactless and noninvasive solution in these oncologically important situations with enormous relevance for patients’ outcomes and prognosis.

By using HSI, spatial and spectral information are obtained from the object under investigation. The resulting three-dimensional datasets are the so-called hypercubes [14]. For this purpose, the contactless illumination of the tissue with light from the visible and near-infrared spectrum takes place at first. Subsequently, the light remitted by the tissue is measured. Based on the resulting spectra, various tissue parameters can be calculated. Because each tissue has different spectral properties, the calculated tissue parameters can be used to differentiate specific tissues’ properties or to analyze tissue perfusion [15]. For example, gastrointestinal cancer has been successfully detected by using HSI [13, 16]. Additional successes have been reported in human oncology for detecting carcinomas and assessment of resection margins, especially in colon cancer [16], thyroid and salivary glands cancer [17], breast cancer [18–20], oral cancer [21], head and neck cancer [22], and brain tumors [23].

HSI, in combination with machine learning algorithms, is intended to automate the visualization and classification of cancer cells. Human studies have already been conducted in gastrointestinal cancer [24], breast cancer [18], skin tumors [25, 26], head and neck cancer [27], and brain tumors [28]. Despite these advances, it is far from routine clinical use. Only a few previous works have dealt with the detection of esophageal cancer and carcinomas of the EGJ using HIS, so far [29–35]. The focus is on automatic tumor detection using HSI during endoscopy based on endoscopic images or tumor detection in histopathological examinations. An application directly on the surgical situs and the specimens during or after preparation and dissection in the OR can only be found in the own study of Maktabi et al. so far [32], using an extraluminal approach of esophagogastric resectates.

In our current study, we aimed to detect carcinomas of the esophagus and the EGJ intraluminally, ex vivo using esophagogastric specimens. To our knowledge, this is the first work to focus exclusively on intraluminal esophagogastric tumor detection using HSI. We intended to classify tumors of the esophagus, the EGJ and the stomach in different tumor stages by HSI. In addition, we aimed to differentiate carcinoma from healthy (mucosal) tissue, thereby demonstrating the potential of HSI for automatic tumor detection.

## Material and methods

### Patient cohort

In this prospective, non-randomized study, all patients with histologically secured carcinomas of the esophagus and the EGJ, who underwent surgical resection at the University Hospital of Leipzig in Germany (from January 2020 until November 2021) and gave their informed consent, were included. The study was approved by the local ethical committee of the Medical School of the University of Leipzig (026/18-ek, 22 February 2018) and was registered at Clinicaltrials.gov (accessed on February 22nd, 2020) (NCT04230603). In addition, and for better understanding of the current analyses, nonselected gastric adenocarcinoma surgical specimens were also examined during this period. A total of 58 patients (12% females) were included, with an average age of 62.8 ± 11.8 years at the time of surgery. Patients had received a neoadjuvant radiochemotherapy, chemotherapy or no neoadjuvant therapy, depending on the preoperative histologically confirmed tumor entity and the c (clinical) TNM classification before first treatment according to the current national and international guidelines. Details of clinicopathological data are provided in Table 1. The surgical procedure also depended on the tumor type and stage, as well as on the localization within the esophagus or the EGJ (according to Siewert’s classification).

**Table 1:** Details of the dataset. Summary of patients’ characteristics, neoadjuvant therapy, and tumor entity

Characteristics	Numbers (%)
Patients	58
Males	51 (88)
Females	7 (12)
Average age (years)	62.8 ± 11.8
*Histopathologic tumor entity*	
Squamous cell cancer	5 (8.6)
Adenocarcinoma	53 (91.4)
*Tumor stage based on TNM classification*	
- ypT0	4 (7)
- pT1/ ypT1	13 (22.4)
- pT2/ ypT2	14 (24.1)
- pT3/ ypT3	26 (44.8)
- pT4/ ypT4	1 (1.7)
*Type of neoadjuvant treatment*	
-Chemotherapy	37 (63.8)
-Radiochemotherapy	13 (22.4)
-none	8 (13.8)

y-neoadjuvant therapy, p-classification provided by histopathologic examination of a surgical specimen, and T-extent of the primary tumor

The HSI technology was included during the operation and in the OR. After complete oncologic resection with systematic lymph node dissection, the specimen was opened lengthwise and prepared on a side-table in the OR. In a separate room, the standardized HSI of the tumor-carrying specimen was taken on the mucosal side within a few seconds. In selected cases, a ruler or tweezers were added by the surgeon to mark the center of the visible tumor (at the site of the mucosa, intraluminally), especially in very small cancers and/or after neoadjuvant treatment and downsizing of the tumor.

### Patient data recording

The HSI cubes were recorded by using a HSI camera of the company Diaspective Vision GmbH (Am Salzhaff, Germany). This camera system records images with 480 × 640 pixels and 100 spectral channels from 500 to 1000 nm. During the recording of the images, all lights in the OR were switched off. RGB data for the annotation were generated using the HSI cube. An expert pathologist (K.S.), together with experienced surgeons (B.J.-W., I.G., C.H.), marked cancerous tissue, as well as healthy esophageal and gastric tissue.

### Data preprocessing

Data were smoothed and normalized by using a median filter and the standard normal variance. Physiological parameters, like tissue oxygenation (StO2), near-infrared perfusion index (NIR-PI), tissue water index (TWI), and the organ hemoglobin index (OHI), were calculated based on the publication of Holmer et al. [36].

### Model

As models, a hybrid 3D-1D-CNN and an inception-based approach with several patch sizes (e.g., 3 × 3, 5 × 5) as input data were tested. These models were tested, because they had already shown good performance in other studies [37]. The best model was selected using a patch size of 3 × 3, a batch size of 128, a learning rate of 0.0001, and a delta optimizer. Hyperparameter optimization by using Bayesian optimization was used to get the best parameters. Early stopping was performed by using the F1-score for cancerous tissue. The hybrid 3D-1D-CNN consists of two-layer Conv3D, two-layer Conv1D, a flattened layer, and a dropout layer. As an activation function, a ReLU was used. In total, 2,182,695 spectra of gastric, 1,467,937 spectra of esophageal, and 295,025 spectra of cancerous tissue were used for modeling. Due to the imbalance of the dataset, class weights were used during the training.

### Metrics

Several metrics were used to analyze the performance of the detection of the three classes. All metrics can be calculated using a confusion matrix. It compares the predictions of the model with the true values. The sensitivity, specificity, F1-score, and the Matthew correlation coefficient (MCC) were calculated [37]. In addition, receiver operating characteristic (ROC) curves could be used to analyze the ratio of true positive and true negative rates of the model. The ROC curve was then a graphical representation that showed the relationship between sensitivity and specificity for different thresholds for each class. To indicate the performance of the model, the area under this curve (AUC) was calculated. All metrics were calculated patient-wise.

### Statistical tests

A t test was performed to analyze the statistical difference of the physiological parameters between the several tissue types (e.g., cancerous and healthy tissue, tumor stage T1/T2 *versus* tumor stage T3/T4 in carcinomas).

## Results

**Fig. 1:**
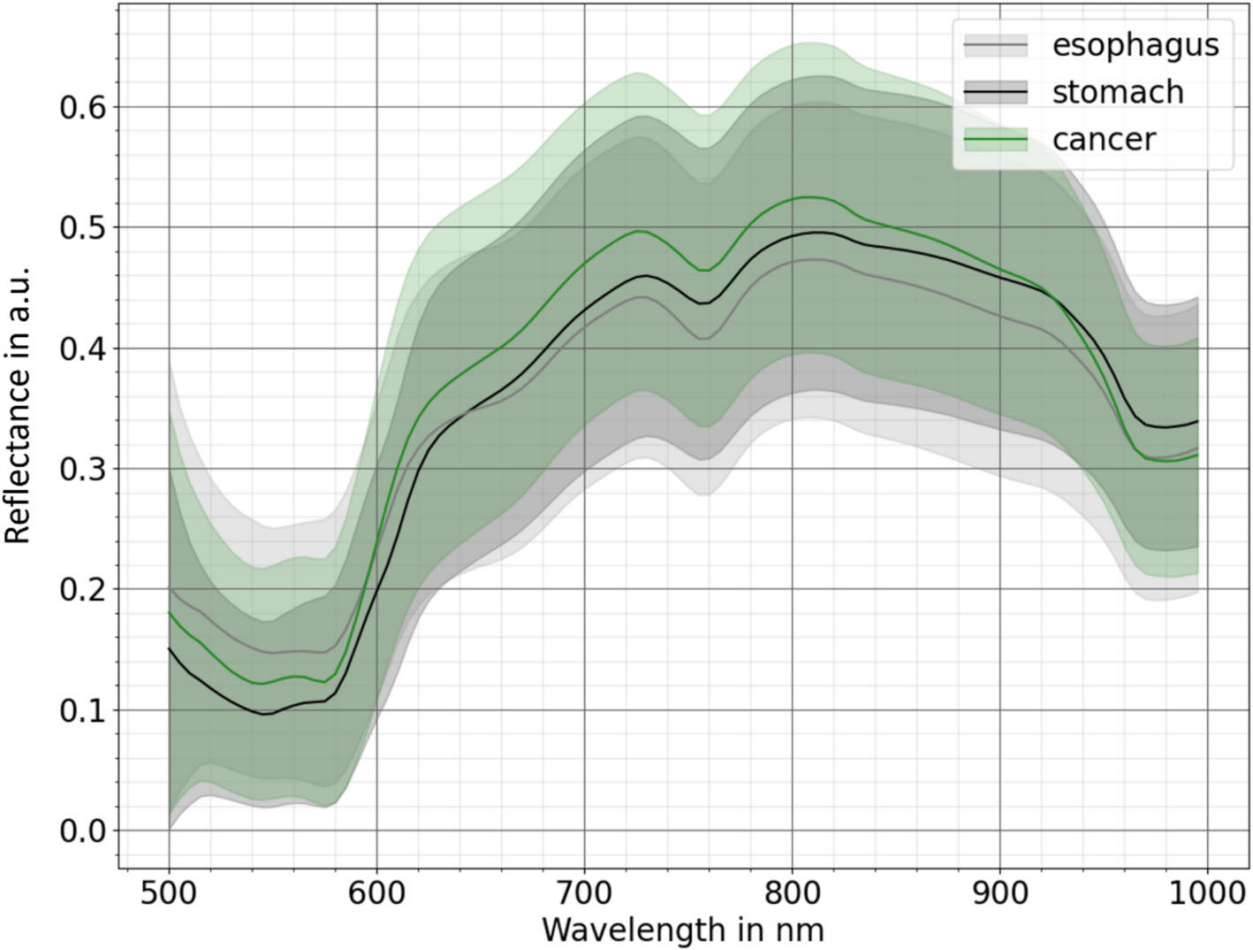
Spectral curves of the dataset without SNV normalization. Solid lines show the mean spectral curves and filled regions standard deviation from the mean curve of the three classes: healthy esophageal tissue (gray), stomach tissue (black), and cancerous tissue (green) (a.u.: arbitrary unit)

A leave-one-out cross-validation was performed, in which one patient was used for testing, three patients for validation, and the other patients for training [37]. The models were trained and evaluated with Python (3.7.4), TensorFlow (2.4.0), on the University of Leipzig cluster with 8 cores of AMD EPYC 32 core processor CPU and 2–8 RXT2080TI GPUs. A pixel-wise classification was done, and only spectra from 520 to 1000 nm were used to reduce noisy input data. Figure 1 depicts the spectra distribution of the three classes. A high standard deviation of all tissue classes is shown. Specific differences in the spectral curves are visible in the water absorption region around 970 nm, as well as in the oxygenation regions (500–600 nm and 750–800 nm) in Fig. 1.

### Classification

**Fig. 2:**
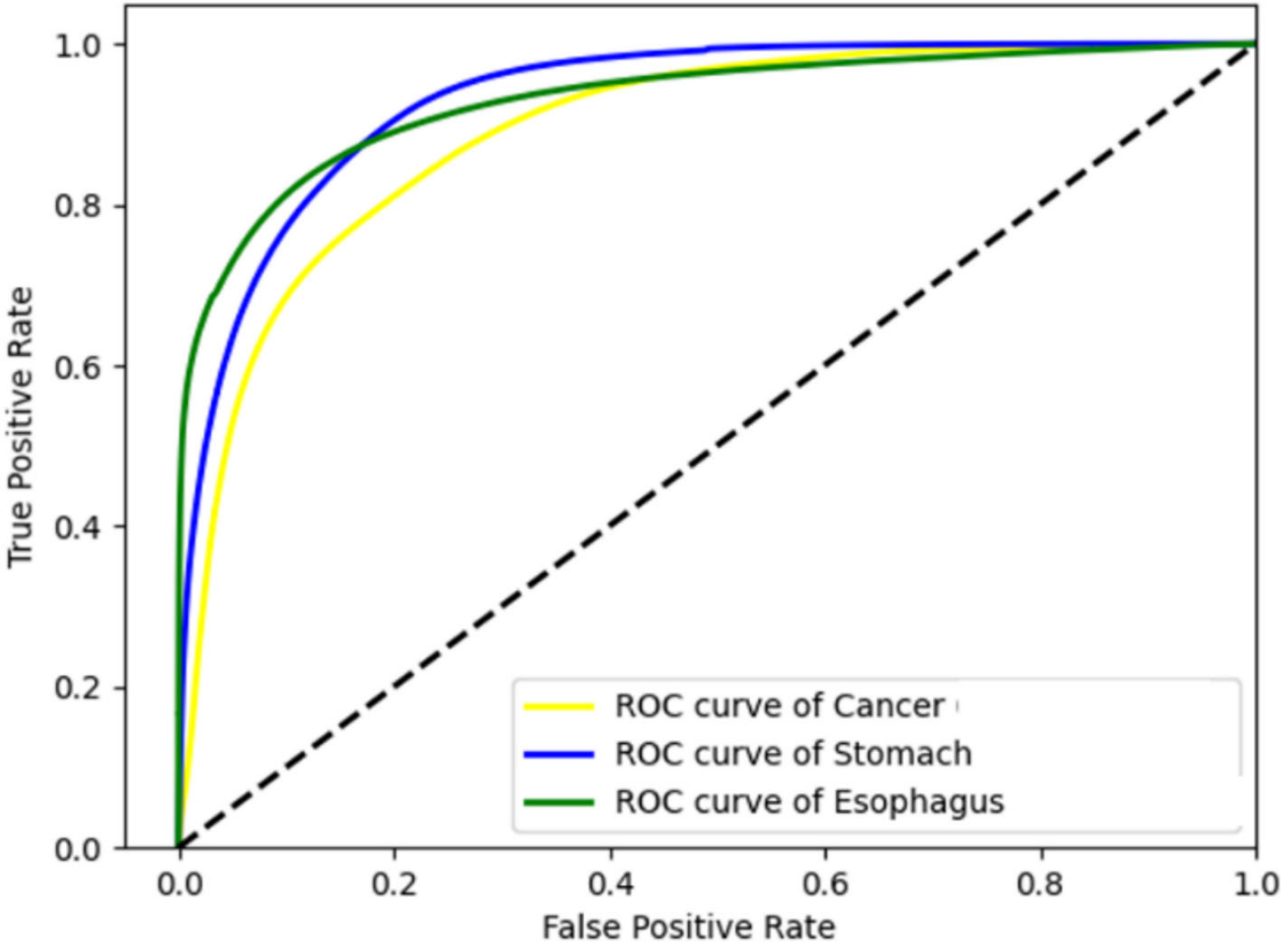
ROC Curves of the three classes cancer (yellow), stomach (blue), and esophagus (green) (3D-1D-CNN)

In Table 2, the best classification results of the leave-one-patient-out cross-validation are shown. A patch size of 3 × 3 by using the 3D-1D-CNN showed the best results. In Fig. 2, the ROC–AUC curve showed the threshold-independent performance of the model. For all metrics, it was noticed that cancerous tissue showed lower values. The standard deviation was for all classes and all metrics at least 0.16 and the highest values were above 0.30 for the sensitivity, F1-score, and MCC of cancerous tissue, as well as the MCC for esophageal tissue. The highest specificity was achieved for gastric tissue with 0.91, and the highest sensitivity was achieved for esophageal tissue with 0.81. The F1-score shows a value below 0.5 for cancerous tissue.

**Table 2:** Averaged performance measurements of the trained model (canctiss: cancerous tissue, stomtiss: healthy stomach tissue, esotiss: healthy esophageal tissue)

Metric	3D-1D-CNN		
			SD
Accuracy	0.79	±	0.2
Sensitivity (canctiss)	0.65	±	0.34
Sensitivity (stomtiss)	0.77	±	0.25
Sensitivity (esotiss)	0.81	±	0.26
Specificity (canctiss)	0.89	±	0.13
Specificity (stomtiss)	0.91	±	0.16
Specificity (esotiss)	0.88	±	0.17
F1-score (canctiss)	0.48	±	0.33
F1-score (stomtiss)	0.8	±	0.22
F1-score (esotiss)	0.84	±	0.23
MCC (canctiss)	0.44	±	0.34
MCC (stomtiss)	0.68	±	0.3
MCC (esotiss)	0.67	±	0.36
AUC (canctiss)	0.8	±	0.16
AUC (stomtiss)	0.79	±	0.31
AUC (esotiss)	0.84	±	0.2

### Visualization

**Fig. 3:**
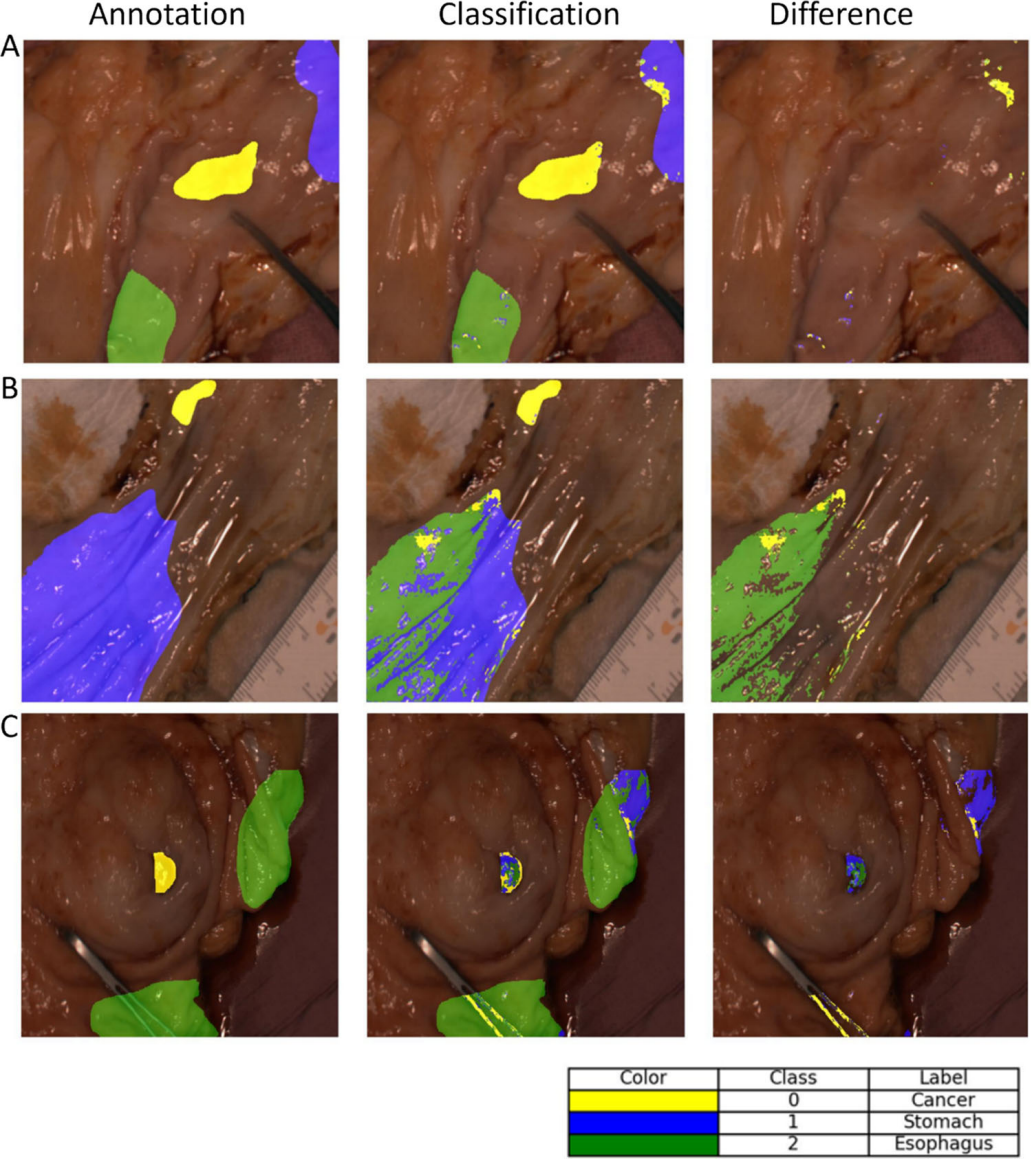
Visualization of the results from the 3D-1DCNN; Patient A showed a very good performance; Patient B showed a false classification of stomach tissue; Patient B showed that the cancerous and esophageal tissue were not well detected

In Fig. 3, the prediction results are demonstrated for two patients by using the 3D-1D-CNN model. The results in Table 2 show a high standard deviation (SD), which is also noticeable in the visualized prediction results. The difference map showed that, especially for the cancer class in C in Fig. 3, the model has problems detecting the whole cancerous tissue. Only partly, the specific tissue was classified correctly. In Fig. 3 (A and B), the difference-map showed good recognition of the cancerous tissue. Section B in Fig. 3 showed that a large part of the stomach tissue was detected as esophageal tissue.

### Physiological parameters

**Fig. 4:**
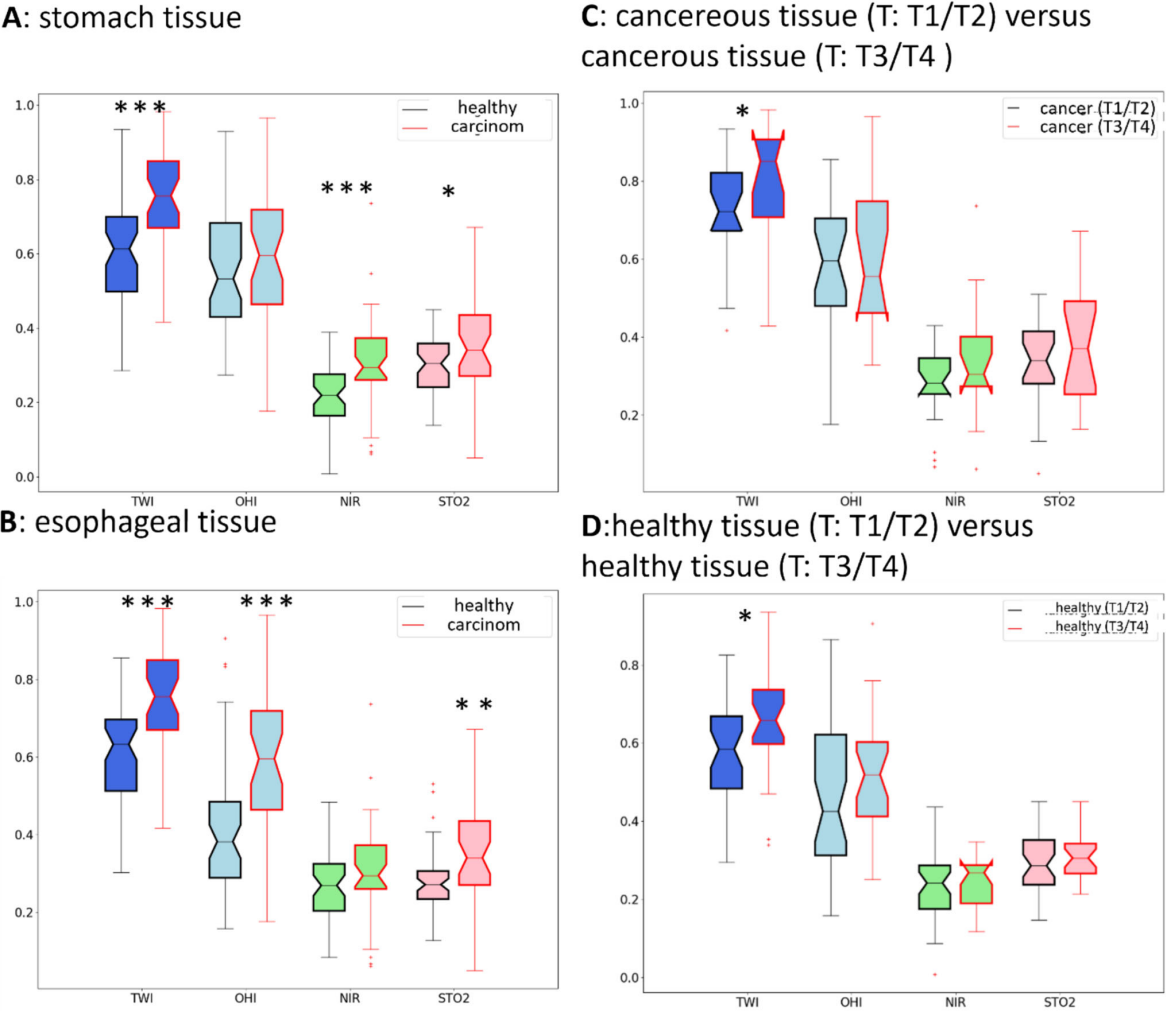
Comparison of physiological parameters (TWI—tissue water index, OHI—organ hemoglobin index, NIR-PI—near-infrared perfusion index, STO2—tissue oxygenation). In A and B, healthy (black) and cancerous tissues (red) are compared from stomach and esophageal tissue. Comparison of tumor stages (T: tumor infiltration) is performed for cancerous tissue in C and healthy tissue in D. Significant differences are marked as *p < 0.05, **p < 0.01, ***p < 0.001

Based on the spectral data, physiological parameters can be calculated. These parameters analyze the tissue regarding water content, oxygenated and deoxygenated hemoglobin. In Fig. 4, it is clearly shown that cancerous tissue had significantly higher water content and oxygen saturation. The hemoglobin content and near-infrared perfusion showed also higher values for cancerous tissue. The tumor stages T3 and T4 had significantly higher water content in healthy and cancerous tissue in comparison with the tumor stages T1 and T2.

## Discussion

Despite significantly improved diagnostics and individualized multimodal therapy options, esophageal carcinomas and adenocarcinomas of the EGJ remain an interdisciplinary challenge. This emphasizes the need for an adjunctive technique that effectively complements existing methods, both for diagnostic purposes and for pre- and intraoperative visualization of tumor extent and margins.

We clearly showed in our study that HSI in combination with AI enables differentiation between healthy mucosa and carcinomatous tissue, both in the esophagus and in the EGJ/the stomach. HSI images of 58 patients with ESCC, EAC, and gastric cancer could be examined.

The results of the classification show that it is possible to distinguish between the different classes. A mean sensitivity of 0.74 for all three classes and a mean specificity of 0.89 were achieved by our current analysis. However, it should be mentioned that the detection of tumor structures reached a lower F1-score and MCC values. This can be explained by the fact that this class was less represented. An improvement in the classification results can be expected, if augmentation techniques are used to increase the number of underrepresented classes. Moreover, we clearly demonstrated that the detection of the cancerous tissue achieved lower sensitivity, especially in the case of ESCC and in dependence on neoadjuvant therapy. It can be assumed that an even distribution of different tumor types and treatment procedures can achieve more robust models for clinical practice.

We also illustrated that, in contrast to healthy mucosa, carcinomatous tissue presents with an increased blood flow by means of HSI measurements. This can be explained by tumor angiogenesis, a process in which the tumor stimulates the formation of new blood vessels by releasing the growth factor VEGF (vascular endothelium growth factor). This process has already been established in the current literature for carcinomas of the esophagus, the EGJ, and the stomach [38–40].

In addition, it has been proven that all tumor stages of all carcinomas examined in our study had a significantly increased water content compared to healthy tissue by HSI. In advanced tumors compared to early tumor stages, this phenomenon was displayed, as well. This finding could be explained by the fact that the area around the carcinoma might express an increased inflammatory reaction with a resulting tendency to edema and, thus, increased water retention. The healthy tissue of advanced tumors also shows an increased water content compared to the tissue of early carcinomas, which could also be related to this thesis. We emphasize that this effect can be caused by neoadjuvant radiochemotherapy, not by chemotherapy. In further studies, the perfusion and physiological parameters should be analyzed based on having a bigger dataset with equally distributed data regarding neoadjuvant therapy and tumor stage.

With HSI, differences in the physiological tissue parameters of hemoglobin and water content can be exhibited in healthy tissue compared to carcinomas, which clearly underlines the large potential of this new imaging modality.

Jansen-Winkeln et al. and Collins et al. have already proven that carcinoma tissue of the esophagus and colon has an increased hemoglobin content compared to healthy tissue [29, 41], which is confirmed by our current study. Likewise, the differences in water content could be validated by us.

Our classification results depicted a high standard deviation. In comparison with the findings of the study of Collins et al. [29], it was relatively high despite the fact that our model was trained on a larger dataset. Probably, the dataset itself plays a significant role here. Firstly, we had a very unbalanced dataset in terms of tissue type classes. Additionally, there was a skewed distribution regarding neoadjuvant therapy with 86% of cases receiving it and only 14% not. The cancer types were also quite heterogeneous. Future studies should aim to include a more balanced representation of cancer types and neoadjuvant therapy cases to improve model performance. To address the class imbalance in our current study, we applied class weighting. While this approach was also used by Collins et al. [29], their results showed a lower standard deviation—approximately half of what we observed. The study conducted by Collins et al. [29] used a binary classification instead of a multi-class classification like we did. The results displayed that the model had difficulties distinguishing between gastric and esophageal tissue (see Fig. 3). Hence, it can be assumed that with our dataset, a binary classification would also show low variance in the classification results. Nevertheless, future work should explore augmentation techniques capable of generating realistic synthetic data to further enhance model robustness. In our study, we tested patch sizes of 3 × 3 and 5 × 5. It can be assumed that larger patch sizes or alternative approaches, such as superpixels, would yield better results. This was already shown by Seidlitz et al. [42], and should be analyzed in detail in future studies.

One of the earliest studies on endoluminal tumor detection of the esophagus using HSI was reported by Seibel et al. [33]. Detection was carried out by combining HSI and fluorescence imaging. This has already been used for tumor detection of different carcinomas. The disadvantage of this method is that fluorescent dye is needed, which has an allergic potential and bears the risk of cardiovascular complications. HSI, on the other hand, is contactless, noninvasively, and does not require a contrast agent.

The disadvantage of HSI is the limitation of the penetration depth. However, data of 58 patients were included in our study only, with consecutive limited numbers of subgroups of different tumor entities and pretreatments. In further analyses, far-reaching methods, such as transfer learning, should be used to investigate whether better performance of the models can be achieved. It should also be emphasized here that we have evaluated intraluminal recordings, and these could currently be recorded ex vivo, only. In future developments, hyperspectral technologies should be integrated into flexible endoscopes or capsule endoscopic systems. This would enable the practical implementation of the proposed approach that combines HSI with AI methods.

HSI has become the focus of medical research in recent years due to its excellent properties and easy application, e.g., in the operating room during surgery. Contactless, noninvasively and without any radiation exposure, the operation process is only minimally disturbed due to the short recording and processing time required intraoperatively. Complications, such as contamination or patient-related adverse effects, are not expected to our knowledge and experience.

## Declarations

### Human and animal participate

The authors declare that all human and animal studies have been approved and performed in accordance with ethical standards.
